# Computational electrophysiology of the coronary sinus branches based on electro-anatomical mapping for the prediction of the latest activated region

**DOI:** 10.1007/s11517-022-02610-3

**Published:** 2022-06-21

**Authors:** Christian Vergara, Simone Stella, Massimiliano Maines, Pasquale Claudio Africa, Domenico Catanzariti, Cristina Demattè, Maurizio Centonze, Fabio Nobile, Alfio Quarteroni, Maurizio Del Greco

**Affiliations:** 1 LABS, Dipartimento Di Chimica, Materiali E Ingegneria Chimica “Giulio Natta”, Politecnico Di Milano, Piazza Leonardo da Vinci 32, 20233 Milan, Italy; 2 Dipartimento Di Matematica, MOX, Politecnico Di Milano, Piazza Leonardo da Vinci 32, 20233 Milan, Italy; 3 Department of Cardiology, S. Maria del Carmine Hospital, corso Verona 4, 38068 Rovereto, TN Italy; 4 U.O. Di Radiologia Di Borgo-Pergine, Borgo Valsugana Hospital, viale Vicenza 9, 38051 Borgo Valsugana, (TN) Italy; 5 Institute of Mathematics, CSQI, École Polytechnique Fédérale de Lausanne, Route Cantonale, 1015 Lausanne, Switzerland; 6 Dipartimento Di Matematica, MOX, Politecnico Di Milano, Piazza Leonardo da Vinci 32, 20233 Milan, Italy; 7 Institute of Mathematics, École Polytechnique Fédérale de Lausanne, Lausanne, Switzerland

**Keywords:** Latest electrically activated segment, Computational models, Epicardial veins, Coronary sinus, Cardiac resynchronization therapy

## Abstract

This work dealt with the assessment of a computational tool to estimate the electrical activation in the left ventricle focusing on the latest electrically activated segment (LEAS) in patients with left bundle branch block and possible myocardial fibrosis. We considered the Eikonal-diffusion equation and to recover the electrical activation maps in the myocardium. The model was calibrated by using activation times acquired in the coronary sinus (CS) branches or in the CS solely with an electroanatomic mapping system (EAMS) during cardiac resynchronization therapy (CRT). We applied our computational tool to ten patients founding an excellent accordance with EAMS measures; in particular, the error for LEAS location was less than 4 mm. We also calibrated our model using only information in the CS, still obtaining an excellent agreement with the measured LEAS. The proposed tool was able to accurately reproduce the electrical activation maps and in particular LEAS location in the CS branches, with an almost real-time computational effort, regardless of the presence of myocardial fibrosis, even when information only at CS was used to calibrate the model. This could be useful in the clinical practice since LEAS is often used as a target site for the left lead placement during CRT.

**Graphical abstract:**

Overall picture of the computational pipeline for the estimation of LEAS

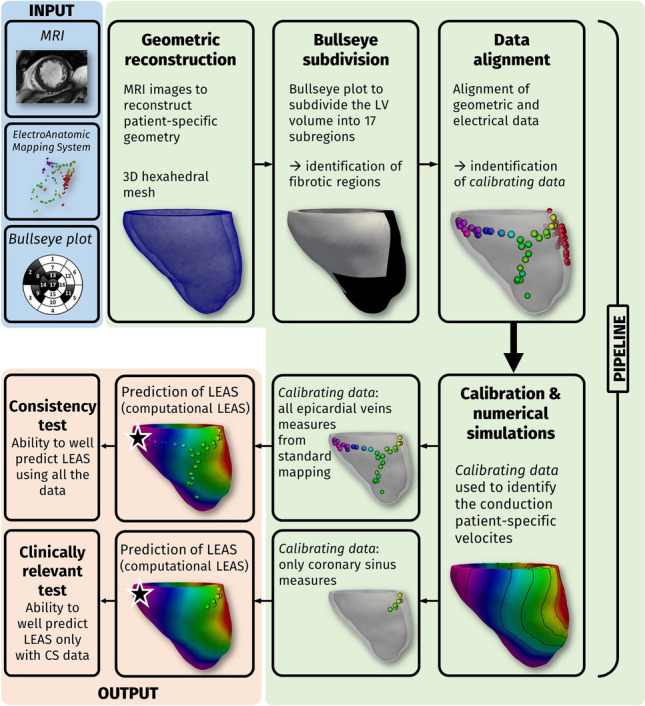

## Introduction

Computational models are nowadays a powerful tool to reproduce and investigate the physiological and pathological electrical activity of the heart with a reduced degree of invasiveness [1–6]. For example, they could be in principle used to accurately reproduce the complete electrical activation of the left ventricle (LV) or of some specific regions, to locate, e.g., the latest electrically activated segment (LEAS). This could be useful for example in the context of cardiac resynchronization therapy (CRT), a therapy which consists in stimulating the LV by means of two electrodes implanted on catheters with the aim of restoring the synchronism of the contraction in dyssynchronous hearts. Indeed, the position of the lead in the coronary sinus (CS) branches is related to the efficacy in restoring the cardiac synchronism, and it is often selected according to LEAS [7–10].

However, in patients with intraventricular conduction disturbances (such as Left Bundle Branch Block, LBBB), direct measures of LEAS require a careful epicardial veins mapping by standard transvenous approach even using an electroanatomic mapping system (EAMS) [11–14]. The acquirement of CS branches activation mapping could be rather laborious and time consuming. For such reasons, the use of computational models could be proposed as an alternative or a support to EAMS. However, the calibration of the model for the patient at hand is still a challenging issue.

In the present work, a computational tool [15] has been proposed for the estimation of LEAS. Our approach allowed us to obtain activation times in all the LV myocardium, providing, in particular, a complete “virtual mapping” of the epicardial veins. In particular, the computational model, based on the Eikonal-diffusion problem, was calibrated by using epicardial activation measures in the coronary sinus (CS) branches obtained by EAMS. The aim of the work was to assess the accuracy of estimates provided by the model in patients with LBBB with and without fibrosis. To this aim, we compared activation maps obtained by the computational model with those measured in the CS branches by means of EAMS, with a particular focus on LEAS prediction. Moreover, we have repeated the same procedure to computationally estimate LEAS by calibrating the model only with the most proximal information (i.e., those acquired in the CS or close to it), simulating a scenario where no EAMS was performed in the CS branches, thus reducing the invasiveness of the standard mapping procedure.

The novelties of the paper, in particular with respect to [15], consisted (i) the use of the Eikonal-diffusion model instead of the monodomain one which allowed us to greatly improve the computational effort; (ii) the application of our tools to patients with fibrosis; (iii) the estimation and validation of LEAS which provides a useful information for the clinical practice, e.g., for CRT; and (iv) the proof that EAMS performed only in the CS is enough to well calibrate the patient-specific model and predict the corresponding electrical activation. This study provides a first necessary step towards the construction of a way to estimate the electrical activity of the ventricles (in particular LEAS) with a reduced electrical mapping and with almost real-time computational efforts. This could be of particular interest for those Electro-Physiology Laboratories (EPLabs) where EAMS is used, for example, to optimize the CRT device.

## Methods

This study was independent, non-industry sponsored, and approved by the local ethical committee.

### Patient-specific geometric reconstruction

Ten patients (P1-P10) affected by severe LV disfunction with LBBB and who underwent EAMS and CRT implant were selected for this study. The etiology of LV dysfunction was ischemic in 4 patients and non-ischemic in 6 patients (2 exotoxic, 1 valvular, and 3 idiopathic). In our electrophysiology laboratory, all patients with indications for ventricular stimulation are implanted with this technique; the criteria for inclusion and exclusion of the implant are those of the guidelines in force. Mean and SD of LV ejection fraction are 28 ± 4%, whereas of QRS width is 139 ± 28 ms.

Patients underwent a comprehensive cardiac MR examination, which included post-gadolinium chelate phase sensitive inversion recovery (PSIR) sequence and pre- and post-gadolinium chelate administration cine balanced steady-state free precession (Cine-SSFP) short-axis sequences. The Cine-SSFP was acquired with a resolution of 2.34 × 2.34 mm^2^ and slice thickness of 6 mm; this sequence is the same as what we adopt in clinical practice: its spatial resolution essentially depends on the slice thickness, which cannot be reduced in order not to lower the signal-to-noise ratio (SNR) and the contrast-to-noise ratio (CNR). In our study, we always found a perfect correspondence between the ventricular fibrosis identified with the 2D SSFP and PSIR sequences. The contrast-enhanced Cine-SSFP images were obtained for all patients with the same parameters and in the same location as for the unenhanced ones.

LV epicardium and endocardium surfaces were segmented using the open-source software MITK (http://www.mitk.org/wiki/MITK). A 3D interpolation has been applied to the short-axis images.

**Fig. 1:**
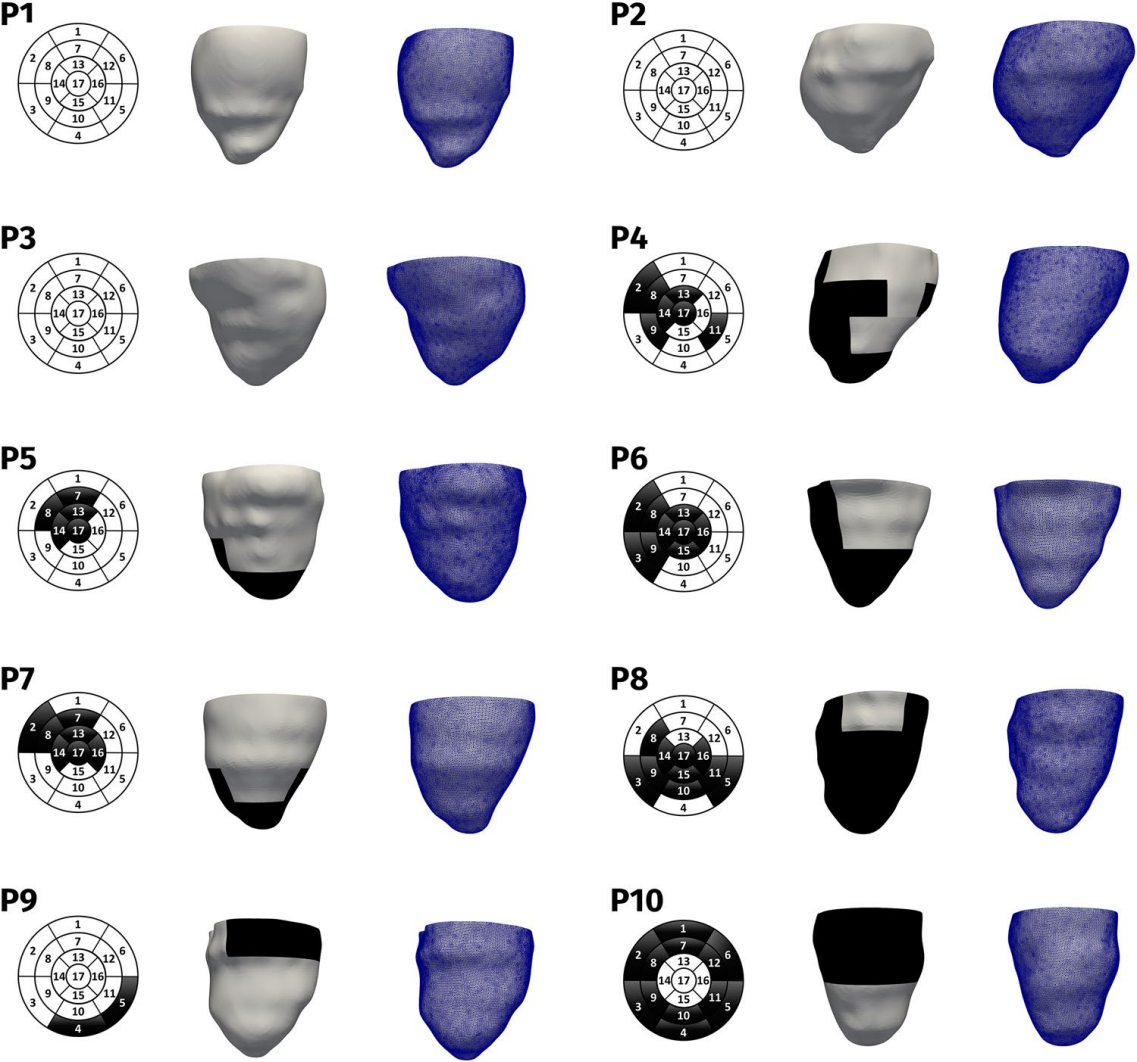
Left: bull’s eye plot of the fibrotic distribution. Middle: front perspective of the reconstructed geometry subdivided into fibrosis (black) and healthy tissue (white). Right: computational mesh

Using suitable *in-house* meshing tools [16], we generated ten finite elements hexahedral volumetric meshes with average cell size of 0.8 mm for each patient (Fig. 1, right).

### Bull’s eye division

The presence of fibrosis was revealed by PSIR and post-contrast Cine-SSFP images in seven patients (P4-P10, degree of fibrosis > 50% for P4-P7, presence of segments with degree of fibrosis < 50% or diffuse intramyocardial pattern for P8-P10). Owing to the T1 component of the signal, post-contrast Cine-SSFP sequence was evaluated in comparison with late gadolinium-enhanced PSIR sequence for revealing the fibrosis [17, 18]. Because of the Cine-SSFP low resolution, we could not properly reconstruct the fibrosis anatomy. However, starting from the standard 17-segment bull’s eye plots built by radiologists with the fibrosis distribution (Fig. 1, left), we developed a tool able to split the reconstructed 3D geometry into 17 sub-volumes representing such segments (Fig. 1, middle). Briefly, the tool follows three steps: projection of the bull’s eye plot onto the endocardium of the reconstructed LV geometry; extension of the projection to the epicardium, obtaining a 3D subdivision of the computational geometry; tagging procedure of each sub-volume into fibrotic and non-fibrotic.

### Electrical data and geometric alignment of computational geometry

For each patient, an electroanatomic mapping of the coronary epicardial veins, in particular of CS branches, was performed by means of the *EnSite Precision* system [19] to record local activation times during the procedure of CRT as previously reported [11–14]. The acquisitions were unipolar and performed over several heartbeats with the same rhythm and morphology, thus excluding anomalous rhythms. Signals were aligned according to the procedure described in [13].

LEAS was defined as a region of the LV with a delay greater than 80% of the total QRS duration. In particular, we were here mainly interested in the point with the absolute maximum activation within this segment (for the sake of simplicity, with an abuse of notation, also referred to as LEAS). In Table 1, the number of total measures N_*TOT*_ has been reported. Moreover, we had also at disposal a mapping of the septum for P1-P5. Notice that the points at the septum were acquired from the RV endocardium, whereas the other ones through a transthoracic procedure.

**Table 1:** Number of total (N_*TOT*_) and coronary sinus (N_*CS*_) measurements used for calibration; conduction velocities $C{V}_{f}$, $C{V}_{s}$, $C{V}_{n}$; mean relative error with standard deviation; distance *D* between measured and computational LEAS. In the first value for each box: Test I (consistency test). In the second value for each box: Test II (towards a clinically relevant test)

	N_*TOT*_/N_*CS*_	$C{V}_{f}$(ms^−1^)	$C{V}_{s}$(ms^−1^)	$C{V}_{n}$(ms^−1^)	Mean relative error (%)	Std (%)	*D* (cm)
P1	39/8	0.71/0.75	0.43/0.45	0.23/0.25	3.53/3.67	2.02/2.12	0.26/0.26
P2	32/7	0.59/0.64	0.37/0.40	0.17/0.19	4.92/5.18	2.65/2.73	0.32/0.31
P3	33/8	0.62/0.64	0.39/0.39	0.19/0.19	5.35/5.92	1.96/1.97	0.21/0.21
P4	32/7	0.61/0.62	0.38/0.39	0.19/0.19	5.62/6.21	2.19/2.35	0.16/0.16
P5	84/10	0.59/0.62	0.38/0.39	0.18/0.19	4.54/5.35	1.83/2.08	0.11/0.12
P6	25/6	0.61/0.63	0.39/0.39	0.19/0.19	5.24/5.44	2.28/2.42	0.41/0.41
P7	17/4	0.60/0.62	0.38/0.39	0.19/0.19	5.20/5.83	2.74/3.04	0.26/0.26
P8	20/7	0.55/0.57	0.35/0.36	0.15/0.16	7.76/8.44	2.5/2.96	0.09/0.09
P9	48/11	0.58/0.60	0.36/0.37	0.16/0.17	6.81/6.96	3.06/3.18	0.37/0.38
P10	86/10	0.59/0.62	0.37/0.39	0.19/0.18	3.25/4.07	1.53/1.87	0.08/0.09

**Fig. 2:**
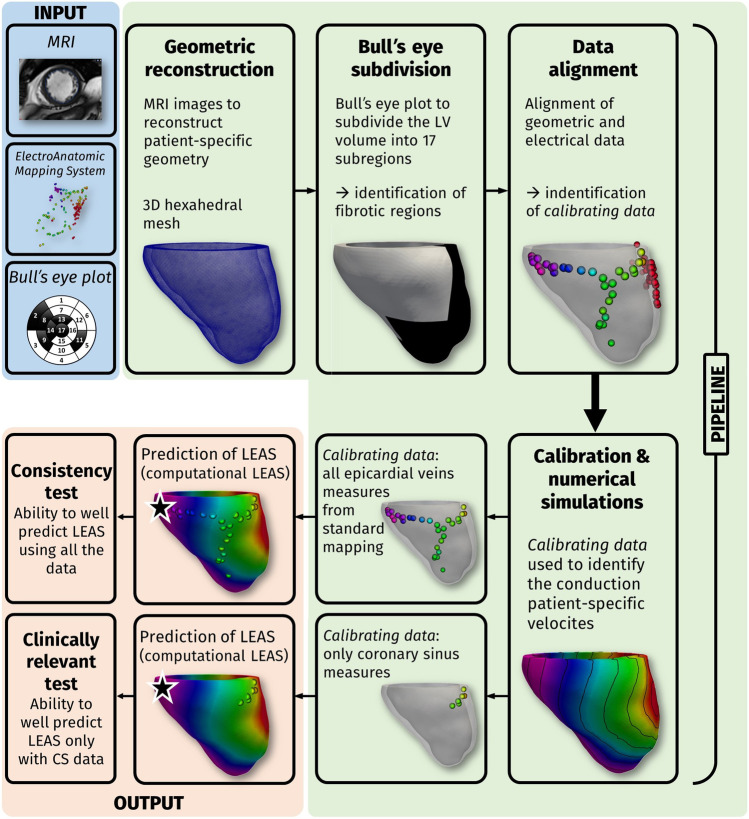
Inputs (in light blue): MRI images, electroanatomic measures, and bull’s eye plots; pipeline steps (in green): geometric reconstruction of the LV; bull’s eye subdivision; alignment of geometric and electrical data; calibration and computational results of activation time; choice of *calibrating data*: (i) all measures at the epicardial veins (Test I); (ii) measures only at the coronary sinus (Test II)

In order to merge electrical and geometric data, we suitably projected all the electrical points on the epicardial surface of the LV geometry properly aligning the measurement point cloud with the geometry and then projecting them onto the reconstructed epicardial surface [3] (Fig. 2).

### Electrophysiological mathematical model

Cardiac electrophysiology (EP) in the LV patient-specific geometries was modeled by using the Eikonal-diffusion equation, which allowed us to obtain the activation maps in the whole LV myocardium [1]:

$${c}_{0}\sqrt{\nabla \psi \frac{\Sigma }{\chi {C}_{m}}\nabla \psi }-\epsilon \nabla \cdot \left(\frac{\Sigma }{\chi {C}_{m}}\nabla \psi \right)=1$$

where $\psi$ is the unknown activation time; $\Sigma ={\upsigma }_{f}f\otimes f+{\upsigma }_{s}s\otimes s+{\upsigma }_{n}n\otimes n$ is the conductivity tensor with ${\upsigma }_{f}$, ${\upsigma }_{s}$, ${\upsigma }_{n}$ the conductivities along the fibers $f$, the sheets $s$, and the normal $n$ directions; $\upchi$ is the surface to volume ratio; ${C}_{m}$ the trans-membrane capacitance; ${c}_{0}$ is a parameter related to the velocity of the depolarization wave along the fiber direction for a planar wavefront; and $\epsilon$ is a dimensionless parameter. For all numerical simulations, we used the following parameters: $\upchi ={10}^{5}{m}^{-1},{C}_{m}=0.01{Sm}^{-2}s,{c}_{0}={0.5s}^{1/2},\upepsilon =0.001$.

We considered an evolutionary version of this model by introducing a pseudo-time variable in order to compute the solution as the steady-state of this modified equation [1]. For time discretization, we used the fully implicit backward Euler method, where the Newton method was applied to linearize the problem at each time step.

We also included in our model the presence of cardiac fibers, according to the Bayer-Blake-Plank-Trayanova rule-based algorithm [20], using the following boundary values for the fibers ($\alpha )$ and the sheets angles $(\beta )$: ${\alpha }_{\text{epi}}=-{60}^{\circ }$, ${\alpha }_{\text{endo}}={60}^{\circ }$, ${\beta }_{\text{epi}}={20}^{\circ }$, and ${\beta }_{\text{endo}}={-20}^{\circ }$.

For space discretization, we used linear finite elements on hexahedral meshes. All the computational frameworks have been implemented in *life*^*x*^ (https://lifex.gitlab.io/lifex), a high-performance C +  + finite element library mainly focused on cardiac applications, based on the deal.II core [21].

When available (P1-P5), the septal data were used as input in the computational simulations. For the other cases (P6-P10), we prescribed as input the activation time in three selected points of the septum, according to standard observations made for LBBB patients [22].

For each patient, the output of the computational simulations was the activation time $\psi$ at mesh vertices.

### Parameter estimation

The activation times measured at the CS branches were used as *calibrating data* to estimate the parameters in the diffusion-Eikonal equation, specifically the conductivities *σ*_*f*_, *σ*_*s*_, *σ*_*n*_. See [3] for further details on the calibration procedure. The conductivity values were differentiated to account for the different velocity of propagation in the three directions. For patients without fibrosis, these values were assumed to be constant in the whole myocardium (although different), whereas for patients with fibrosis, we calibrated two values of conductivity for each direction, one for the healthy tissue and one for the fibrotic segment, where conductivity is expected to be reduced but not necessarily zero as happens in presence of scars [23]. In order to properly select for each patient the values of the conductivities, we minimize the discrepancy at the EAMS points between activation times obtained by computational simulations and those acquired by EAMS. In particular, in a first test, we used all the available data at disposal applying a basic direct search method inspired by the Hooke and Jeeves pattern search optimization method [24], which does not require the computation of a gradient (*consistency test*). We used the procedure described in [3], based on iteratively solving the Eikonal-diffusion problem with updated values of conductivities, starting from an initial guess in physiological ranges and on ongoing corrections given by the solution of the Eikonal-diffusion problem itself. In particular, for patients with fibrosis, we looked for two values of the conduction velocity (one in the healthy tissue and one in the fibrosis) in order to match the given data. See [3] for further details and for a validation of this approach in the context of the monodomain model.

We also repeated the estimation of conductivities by using as calibrating data only the most proximal measures, i.e., those acquired at the CS or very close to it (*clinically relevant test*). In Table 1, the number of measures N_*CS*_ at CS has been reported.

### Reconstruction of the epicardial veins

We finally needed to reconstruct the epicardial vein geometries, where the computational LEAS was evaluated. The MRI images at our disposal were not fine enough to allow the segmentation of such veins. Thus, we proposed here a method for their reconstruction. The idea was to exploit the locations of the points acquired by EAMS to draw the anatomy of the veins through the use of splines, an accurate mathematical tool widely used for interpolation, e.g., in computer graphics [25]. For this purpose, we used the Paraview software (https://www.paraview.org/), which allowed us to manually manage the control points of the splines to improve the geometric reconstruction of the epicardial veins.

### Test I: Prediction of the latest electrically activation segment: a consistency test

Thanks to the computational pipeline described above and reviewed in Fig. 2, we were able to solve the Eikonal-diffusion problem with the estimated conductivities and to compute for each patient the activation times in the myocardial geometry. In particular, this allowed us to identify the *computational* LEAS, that is, the point in the reconstructed veins featuring the latest activation segment among all the ones obtained by the computational simulations, and to compare it with the measured LEAS, that is, the one obtained by EAMS. This *consistency test* allowed us to assess the suitability of the computational model to accurately estimating measured LEAS.

### Test II: Prediction of the latest electrically activation segment: towards a clinically relevant test

The previous estimation of computational LEAS and the comparison with measured LEAS have been then repeated in the case when only the most proximal measures, i.e., those at the CS (or very close to it) were used to calibrate the model, see Fig. 2 (*clinically relevant test*). In this case, we proposed to verify if our method was able to predict LEAS well by using only few data, in particular those at CS. This could provide a way to predict LEAS by using a shorter mapping procedure than the standard one. In particular, it might be enough to map only the most accessible epicardial points (i.e., those at CS) to extract clinically relevant information about LEAS.

## Results

### Test I: A consistency test

**Fig. 3:**
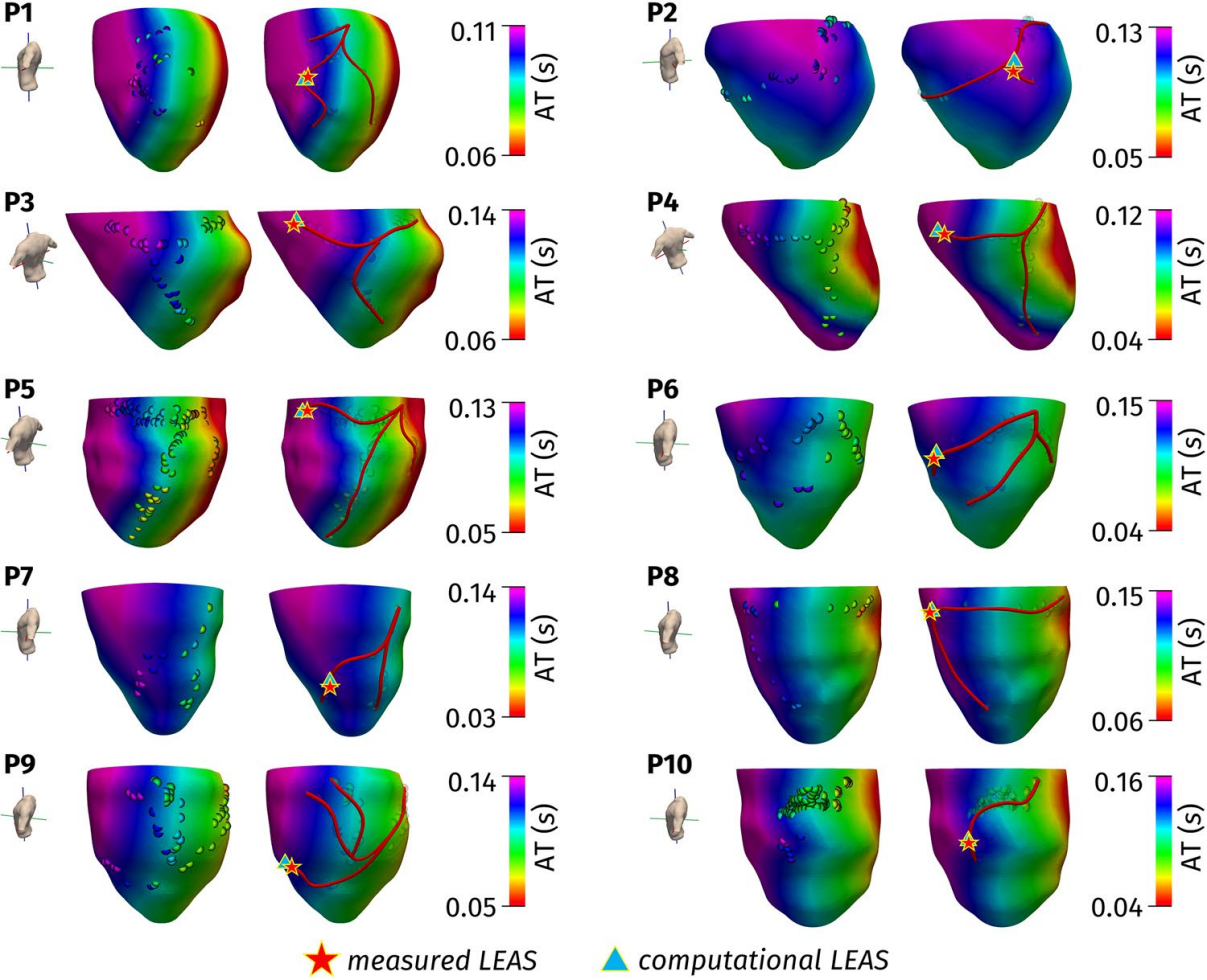
Left: Computed (continuous map) and measured (bullets) activation times. Right: Reconstructed epicardial veins (in red) and location of LEAS. Test I (consistency test)

In Fig. 3, we reported the collection of geometric and electric data (in bullets) after their alignment, together with the continuous maps of activation times obtained by the computational simulations after calibration with all the CS branch measures. The calibrated conductivities lead to physiological conduction velocities for all the patients considered, specifically in the range (0.55–0.75) m/s along the longitudinal direction of the fibers, in the range (0.35–0.45) m/s along the transversal direction and in the range (0.15–0.25) m/s along the normal direction [26]. In particular, in Table 1, we reported the values of the conduction velocities estimated for each patient to match the measures. In the segments characterized by fibrosis, we found for the three conductivities values reduced by about 40% for P4, P6, and P8; about 30% for P5, P7, and P9; and about 50% for P10.

**Fig. 4:**
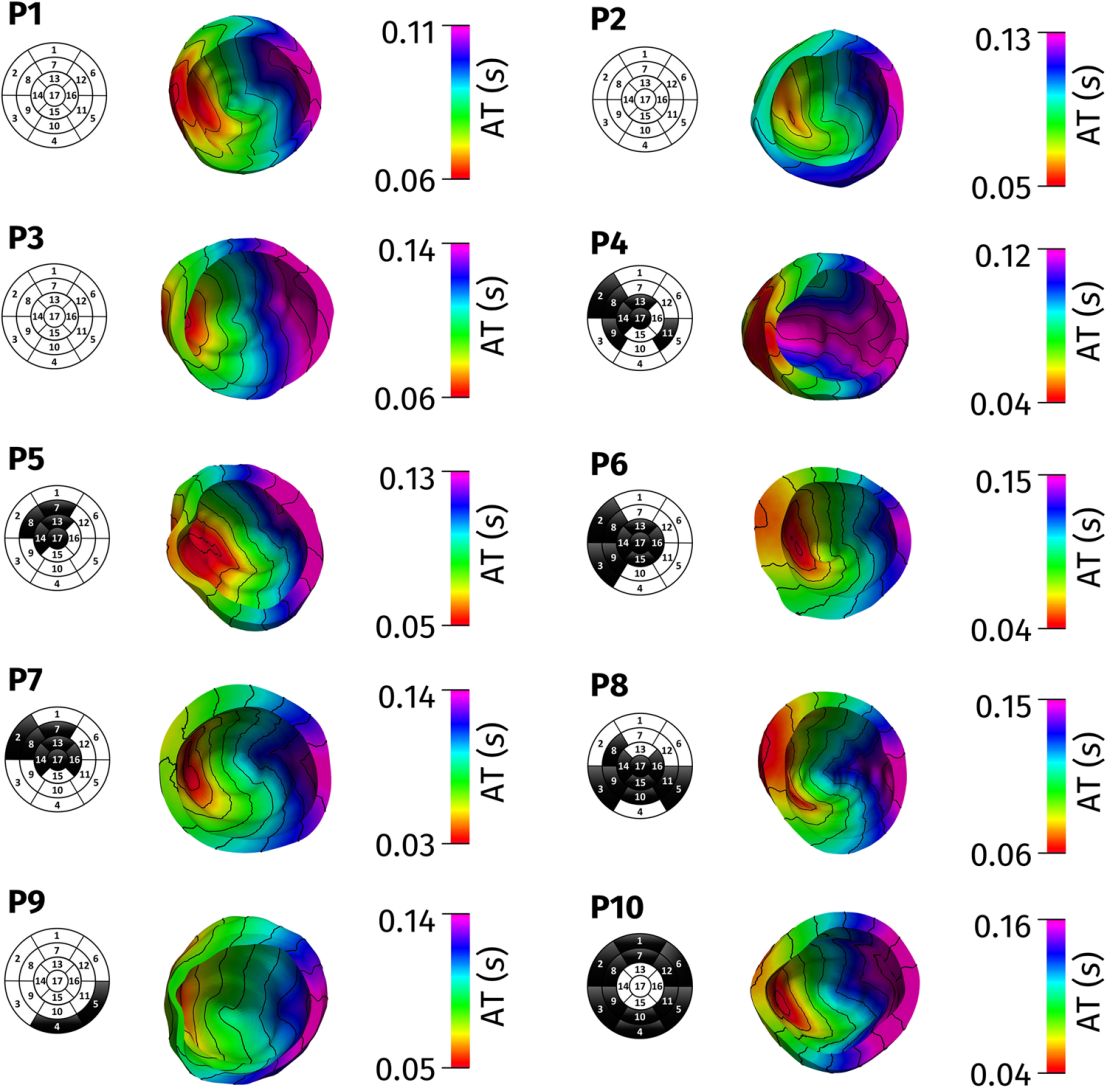
Computed activation times with contour lines together with bull’s eye plots

In Fig. 4, we show the computed activation maps from the base point of view. We can notice that for all the cases, the first activated region is the septum. This is coherent with the electrical propagation in LBBB patients where the electrical signal enters the LV through the septum activated by the right ventricle. Notice from the corresponding bull’s eyes the lower velocity of propagation in the region with fibrosis.

From Fig. 3, we observe a very good agreement between computations and measures. To provide a quantitative analysis, in Table 1, we report the errors obtained by our computational simulations. In particular, we computed the mean relative error and the standard deviation over the total number of measurements. We observe an excellent agreement between computational experiments and measures, the error being in any case less than 8%. In particular, for patients without fibrosis (P1–P3), the average error was 4.6%, whereas for the patients with fibrosis (P4–P10), it was 5.5%. This is not surprising, since in the latter case there are more parameters to determine (the three conductivities being not constant in space).

In Fig. 3, we showed also the reconstructed epicardial veins together with the position of measured LEAS and computational LEAS. We notice an excellent agreement between the positions of the two LEAS.

To go deeper in the analysis, in Table 1, we reported the values of the distance *D* (intended as the geodesic distance over the epicardial surface) between measured and computational LEAS. These results confirmed the great ability of the computational tool in predicting LEAS, the error in terms of distance being in any case less than 0.4 cm (average 0.23 cm). *D* assumed an average value of 0.26 cm for P1–P3 (that is, for patients without fibrosis) and of 0.21 cm for P4–P10 (that is, for patients with fibrosis), highlighting that the accuracy of the computational tool in predicting the location of LEAS is independent of the presence of fibrosis.

### Test II: Towards a clinically relevant test

**Fig. 5:**
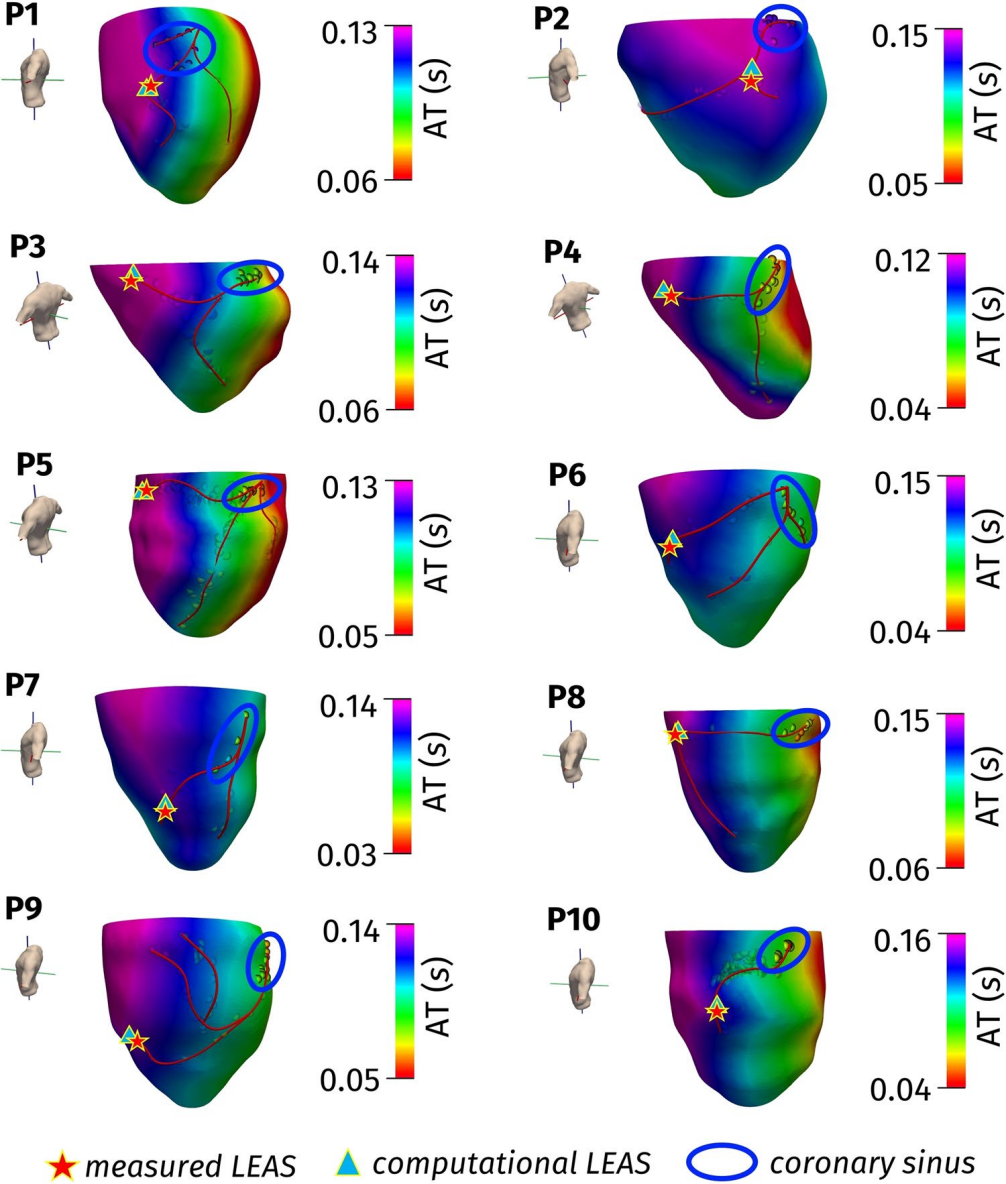
Computed (continuous map) and measured activation times in the coronary sinus (rounded in blue), reconstructed epicardial veins (in red) and locations of LEAS. Test II (towards a clinically relevant test)

In the second test, we assessed the accuracy of the computational LEAS predicted by computational simulations calibrated by using only measures acquired at CS, that is, the most proximal ones. In Fig. 5, we reported the corresponding location of LEAS together with the continuous computational map of activation times and the reconstructed epicardial veins.

In Table 1, we reported in the second value for each box the values of the estimated conductivities together with the relative error (obtained against all the measures at disposal, not only those at CS) and the standard deviation for Test II. From these results, we observe that the conductivity values estimated by using only the measures at CS are very similar to those obtained in Test I, where all the measures were used for calibration. Accordingly, we observe a very small increment of the relative error with respect to Test I.

In Table 1, we reported (in the second value for each box) also the values of the distance *D* between computed and measured LEAS defined above. *D* resulted to be comparable to the same quantity obtained for Test I (consistency test).

## Discussion

In this work, we have proposed a computational tool which was applied to 10 LBBB patients with cardiomyopathy (3 without fibrosis, 1 with moderate fibrosis, and 6 with wide ventricular fibrosis) with the aim of predicting the electrical activation maps in the CS branches. The error between computations and measures obtained by EAMS was in any case less than 8% showing the accuracy of our tool, which is able to reproduce a complete myocardial activation map (Fig. 3, left). This could be thought as the first step towards the modeling of the processes that are at the basis of intraventricular conduction disorders. More specifically, we focused on the prediction of LEAS, which has been seen to be an optimal site for the location of the left lead during CRT [7, 11]. Other techniques could be also used to optimize CRT after the implantation, for example echocardiography or other tools to define atrioventricular (AV) and ventriculo-ventricular (VV) delays [27, 28].

Our results showed that the distance between LEAS mapped during EAMS by cardiologists and those predicted by our computational model is in any case less than 4 mm (Table 1), indicating the great ability of our tool to well predict LEAS location. Our results showed to be independent of the presence of fibrosis (P1–P3 vs P4–P10) and of the knowledge of septum data to be used as an input (P1–P5 vs P6–P10).

More interestingly, we showed that it is enough to know the activation times at the most proximal sites (i.e., at the CS) to well predict the location of LEAS distally in the CS branches.

Notice that this is not a statistical study, rather a computational one. This means that we built an a priori model based on the physical principles and not an a posteriori model based on data and measurements as required by statistics. The data (from EAMS) were used to calibrate such model.

For this reason, our sample (10 cases) could not be considered too limited, and it allowed to prove the validity of our model.

Computational methods represent nowadays a very promising, non-invasive tool to provide clinical indications in different applications of electrophysiology. In particular, there is in the literature a growing interest in using computational models to predict and support the clinical practice for CRT [2, 29–35]. Despite the emergence of new and promising techniques such as hisian and left bundle branch pacing, CRT remains the main therapy in patients with ventricular dyssynchrony associated to intraventricular conduction disorder and ventricular dysfunction [36]. However, CRT has a non-responder rate of about 30% [37]. A possible way to improve CRT, in terms of clinical outcome and patient follow-up, consists in the optimal localization of the LV lead. The use of EAMS for the mapping of the CS and its branches can guide CRT implantation by indicating LEAS, which has been seen to be an optimal site for the location of the left lead during CRT [11–14].

Our work could be of utmost importance in view of a possible clinical application, since it gave preliminary positive answers to the question if it is possible to use computational methods to support CRT. Indeed, it showed how it is possible to well predict LEAS, thus giving useful indications about the location of the left lead during CRT, performing only a CS mapping.

Previous studies have suggested that LV pacing in a site with late activation (either mechanical or electrical), rather than anatomically pre-specified left ventricular segments, may improve the hemodynamic response, reverse remodeling, and clinical outcome of patients that underwent CRT implantation [7–10]. In particular, it was demonstrated that there was a strong correlation between CS-LEAS and branches-LEAS: in other words, the vessel with the highest activation delay origins from the CS regions of highest activation delay; this finding can reduce procedural time during CRT implant, with or without EAMS, by limiting the search of the target vessel for LV lead placement to the CS area with the largest delay [38]. The possibility of predicting the site of highest delay in the LV with a mathematical model with the acquisition of a few points in CS, without the need to extend the mapping procedure to the venous branches afferent to it, could facilitate the EAMS, providing a potential benefit for patients. Thus, the exposure time of the patient to the mapping procedure is highly reduced and a great simplification of the procedure is allowed. This may be of some clinical use during CRT implantation.

These promising results are corroborated by the low computational costs of the Eikonal-diffusion model. This allows to describe the spreading of electrical activation with reduced computational times with respect to the monodomain and bidomain models [36, 39, 40]. In the present work, the computational effort of a single numerical simulation was about 30 s. The calibration procedure was composed for each patient of about 7/8 numerical simulations (in average). Then, for each patient, the accurate computation of the activation times of the entire LV and in particular the localization of LEAS took about 4 min. These very low computational times suggested the perspective to investigate the inclusion of our computational tool in an electrophysiology mapping device in order to shorten the invasive procedure (only CS could be needed) and give an effective support in determining activation maps and in particular LEAS position.

Some remarks and limitations are now due. First, we provide in what follows a note on the computational effort of the proposed pipeline. We have stressed that the solution of the Eikonal-diffusion problem and of the minimization algorithm is very cheap. However, we have to account also for the time needed by the pre-processing procedures. In particular, we estimated that the segmentation of MRI images to obtain the computational mesh needed about 20 min per patient. The same amount of time was required for the fibrotic patients to adapt the mesh to account for the presence of fibrosis. The generation of the cardiac fibers required instead a very low computational cost (less than a minute). Notice however that these three procedures could be performed once at all for each patient and thus outside the minimization loop. Also, they could be performed before the electrical mapping procedure since only MRI data (often acquired some days before EAMS) are needed.

A possible limitation is instead given by the time needed to register geometric (MRI) and electrical (EAMS) data. This required about 25/30 min per patient and, unlike the preprocessing procedures described above, could not be performed before EAMS. This would make more difficult the inclusion of our tool in the clinical practice. For this reason, we are working to speed up this procedure in order to make possible a true real-time computation of LEAS.

Another limitation is given by the fact that in Test II, we used the knowledge of the electrical mapping in the CS branches to reconstruct the epicardial veins to be used for the search of LEAS. Of course, for this test, we would not have such information since EAMS would be made only up to the CS. However, notice that our computations, without knowing the location of the epicardial veins, would be able to give an information about LEAS in all the epicardium, and not only in the veins. This information could be useful for the cardiologists who could then locate the left electrode in the vein point closest to this “global” LEAS. In any case, to definitely fix this point, we are currently working on CT images that, unlike MRI, allow to have enough information to segment the epicardial veins, thus overcoming the previous limitation.

## Conclusions

In this work, we have proposed a computational tool for the rapid prediction of myocardial electrical activation maps and in particular of the location of LEAS in the CS branches, usually used for CRT implantation. The model was calibrated by using the activation maps obtained by EAMS navigating CS branches. Its application to ten patients with LBBB and cardiomyopathy with possible fibrosis showed an excellent agreement between computational and measured activation maps and LEAS. Remarkably, we showed that it was enough to use activation maps only at CS to calibrate the model in order to well predict the ventricular electrical activation and in particular LEAS in the CS branches. This could be of particular interest for those EPLabs where EAMS is routinely used, for example in view of the implantation of a CRT device.

These results provide a first preliminary step towards the use of computational tools to better understand the conditions that could lead to intraventricular conduction disorders and to assist CRT by providing a support and simplification of EAMS.

## Data Availability

Data related to computational meshes and numerical results will not be shared.
